# msmsEval: tandem mass spectral quality assignment for high-throughput proteomics

**DOI:** 10.1186/1471-2105-8-51

**Published:** 2007-02-09

**Authors:** Jason WH Wong, Matthew J Sullivan, Hugh M Cartwright, Gerard Cagney

**Affiliations:** 1 Chemistry Department, Oxford University, Physical and Theoretical Chemistry Laboratory, South Parks Road, Oxford OX1 3QZ, UK; 2 Conway Institute, University College Dublin, Belfield, Dublin 4, Republic of Ireland

## Abstract

**Background:**

In proteomics experiments, database-search programs are the method of choice for protein identification from tandem mass spectra. As amino acid sequence databases grow however, computing resources required for these programs have become prohibitive, particularly in searches for modified proteins. Recently, methods to limit the number of spectra to be searched based on spectral quality have been proposed by different research groups, but rankings of spectral quality have thus far been based on arbitrary cut-off values. In this work, we develop a more readily interpretable spectral quality statistic by providing probability values for the likelihood that spectra will be identifiable.

**Results:**

We describe an application, msmsEval, that builds on previous work by statistically modeling the spectral quality discriminant function using a Gaussian mixture model. This allows a researcher to filter spectra based on the probability that a spectrum will ultimately be identified by database searching. We show that spectra that are predicted by msmsEval to be of high quality, yet remain unidentified in standard database searches, are candidates for more intensive search strategies. Using a well studied public dataset we also show that a high proportion (83.9%) of the spectra predicted by msmsEval to be of high quality but that elude standard search strategies, are in fact interpretable.

**Conclusion:**

msmsEval will be useful for high-throughput proteomics projects and is freely available for download from . Supports Windows, Mac OS X and Linux/Unix operating systems.

## Background

The identification of proteins by tandem mass spectrometry (MS/MS) is an important step in many proteomics studies [1]. The introduction of orthogonal peptide separation techniques coupled to the mass spectrometer, such as multidimensional protein identification technology (MudPIT) [2] and combined fractional diagonal chromatography (COFRADIC) [3], has significantly increased the potential throughput of tandem mass spectrometry experiments, enabling the identification of 100s or 1000s of proteins from a single sample. Yet, this potential has not been fully realized because the vast amount of primary data generates computational burdens, notably time-consuming and processor-intensive tandem mass spectra interpretation. The most widely-used interpretation programs, such as SEQUEST [4], X!Tandem [5] and Mascot [6], use amino acid sequence databases that are expanding in size daily. Recently, heuristic programs such as X!Tandem [5] and PFSM [7] have been reported to reduce search times by 80–90%. Even so, an emerging goal for the biologist is to identify the post-translational modifications or mutations in proteins that are often the basis for disease states [1,8]. Search time would grow exponentially if the search space is increased to account for all possible modifications.

One approach for reducing search time is to remove MS/MS spectra that are unlikely to be identified. Currently, up to 90% of tandem mass spectra recorded in a typical multiple dimensional chromatography (LC^*n*^) MS/MS run cannot be identified by database search methods. There are multiple reasons for this, including the presence of non-peptide signals derived from chemical or electronic sources. Of the spectra that do represent peptide signals, many remain unidentified because the spectra are of poor quality, or because the cognate proteins contain amino acid polymorphisms, post-translational modifications, or splice variants that are not anticipated by the search programs. Other proteins are simply not present in the sequence databases.

Recently, attempts have been made to infer spectral quality by combining a finite number of spectral features into a score that discriminates low from high quality spectra [9-14]. In general, the methods rely on combining a finite number of spectral features into a score that is a measure of spectral quality and discriminates high quality from low quality spectra. For discriminant function definition, a training dataset partitioned by cross-correlation score (e.g. [10]) or containing pre-annotated spectra (e.g. [8]) is used. Early efforts focused on defining general discriminant function primarily used for the rejection of low quality spectra prior to database searching [11]. More recently, Nesvizhskii and coworkers (2006) described an approach where a new discriminant function is defined for each dataset for finding high quality spectra that have not been annotated by a first pass database search [12]. Bern and coworkers (2004) also prioritized spectra for intensive interpretation efforts and used regression analysis to generate a continuous score measuring the number of b- and y-ions in a spectrum [9].

Here, we present a statistical model that assesses the quality of tandem mass spectrum from any LC^n^/MS/MS run (or collection of MS/MS spectra) prior to database searching. In previous work, authors have suggested arbitrary cut-off scores that may be used as a guideline to determine whether a spectrum is a likely candidate for further analysis, but such an approach is undesirable because average spectral quality typically varies with different samples, experiment formats, instruments and laboratories. Furthermore, even if the discriminant function has been adapted for a particular dataset, arbitrary cut-off scores are difficult to interpret in relation to their ability to reject low quality spectra. We therefore build on previous efforts and present a freely-available program that assigns the probability that a tandem mass spectrum will yield a positive peptide identification. We show that our assigned probability is indeed a good estimate of the observed value and therefore is of practical use in a proteomics lab using different instrument platforms or different types of experimental samples. msmsEval is useful for reducing search processing time and for selecting high quality unidentified spectra for further assessment.

## Results

### Algorithm development

#### Experimental datasets

Two groups of experimental MS/MS datasets were used to train and evaluate the spectral quality evaluation algorithm.

1) The UCD dataset was generated in-house and consisted of 142,582 MS/MS spectra from 22 LC^*n*^/MS/MS runs, including commercial standard proteins, cultured cell extracts, and human vascular proteins, were acquired from different samples over a 6 month period. Three different database search strategies were used to annotate the dataset.

Briefly, SEQUEST [4]/PeptideProphet [15]/ProteinProphet [16] was used to identify spectra whose annotated amino acid sequences were presented in the UniProtKB/Swiss-Prot database (release 6.0). InsPecT [17] was used to identify peptides based on sequence tags and pepNovo [18]/SPIDER [19] used a combined *de novo*/tag approach (see experimental methods for details). The three strategies represent distinct approaches to identifying peptides from experimental tandem mass spectra (typically only the first method is used in most labs).

If a spectrum is matched by any of the three search methods described, these were regarded as annotated. Of the 142,582 spectra, 16,999 were annotated and therefore classed as identifiable (see Additional file 1). The presence of mislabeled spectra in the training data could degrade the accuracy of prediction of the resulting classifier [20]. To minimize this, we applied a 9-fold cross-validation/k-nearest neighbor strategy (see Additional file 2: Appendix 1).

The 22 runs were randomly divided into training and test datasets such that the training dataset consisted of 12 runs with 9,044 identified and 69,584 unidentified, while the test dataset consisted of 10 runs with 7,955 identified and 55,999 unidentified.

2) *ISB dataset*. The second dataset was the publicly available ISB dataset consisting of 22 LC/MS/MS runs of artificially generated protein mixture digests [21]. We used this dataset to compare identification probabilities predicted using our program to the identification frequencies observed by Tsur and coworkers who used a "blind" approach resulting in the identification of many modified proteins [18]. This dataset consists of 39,408 MS/MS spectra. Initial efforts using SEQUEST by ISB annotated 4,310 spectra, while Tsur and coworkers [18] were able to annotate an additional 1,176 spectra. Note that the number of spectra in the dataset appear to differ from those available from the website. This is because the DTA files generated by Keller and coworkers [21] clustered adjacent MS/MS scans of similar parent mass. Because our algorithm evaluates scans individually without clustering, the spectra annotations from ISB and Tsur and coworkers [18] were "declustered" to generate our dataset.

### Spectral features

A set of spectral features, similar to those used by previous workers, was used to measure the quality of MS/MS spectra. We applied a unique normalization procedure in an attempt to capture specific information from *b *or *y *ions that may be present. The intensity of peaks was initially normalized by assigning to each peak its intensity rank within a local segment, *i*, of 56 m/z for each spectrum, this value being chosen because there normally will be no more than one *y *and *b *ion within such a segment unless the charge state of the precursor ion is 3 or greater. This improves the signal-to-noise ratio for the whole spectrum by taking advantage of the fact that *b *or *y *ions are typically the most intense ions within any local region of a peptide tandem mass spectrum [22]. We tested all features in combination, details are reported in Appendix 2.

In order to eliminate potential feature bias between spectra generated from singly and multiply charged precursors, these were distinguished using a similar algorithm similar to that described by Hansen and co-workers [23], and the maximum *m/z *value, was adjusted to the mass of the precursor ion for spectra derived from singly charged ions. We found no significant performance differences when we streamed our data into +1, +2, and +3 sets, in agreement with previous reports [9,11]. The definition of each spectral feature can be found in the experimental methods section.

### Combining spectral features by logistic regression

The next step is to combine spectral features in order to make predictions on their general quality. Numerous classification algorithms may be used. In general, non-linear classifiers such as quadratic discriminant analysis (QDA) or multi-layered artificial neural networks tend to provide better classification than linear classifiers such as linear discriminant analysis (LDA) if the dataset is not linearly separable. To test whether non-linear classifiers are likely to be advantageous in this case, the training dataset was used to train a quadratic discriminant function and a linear discriminant function. The functions were then evaluated using our UCD test dataset. In this case, no significant difference was observed (see Additional file 2: Appendix 2) and as result a linear classifier was chosen for its simplicity in application and interpretation.

Logistic regression is a method that allows the discrimination between two or more groups of samples based on a vector of given variables for each sample through a logistic function. The basis of the method is similar to that of linear discriminant analysis (LDA) in combining spectral features by a weighted linear function, such that,

D(x1,...,xn)=co+∑i=1ncixi     (1)
 MathType@MTEF@5@5@+=feaafiart1ev1aaatCvAUfKttLearuWrP9MDH5MBPbIqV92AaeXatLxBI9gBaebbnrfifHhDYfgasaacH8akY=wiFfYdH8Gipec8Eeeu0xXdbba9frFj0=OqFfea0dXdd9vqai=hGuQ8kuc9pgc9s8qqaq=dirpe0xb9q8qiLsFr0=vr0=vr0dc8meaabaqaciaacaGaaeqabaqabeGadaaakeaacqWGebarcqGGOaakcqWG4baEdaWgaaWcbaGaeGymaedabeaakiabcYcaSiabc6caUiabc6caUiabc6caUiabcYcaSiabdIha4naaBaaaleaacqWGUbGBaeqaaOGaeiykaKIaeyypa0Jaem4yam2aaSbaaSqaaiabd+gaVbqabaGccqGHRaWkdaaeWbqaaiabdogaJnaaBaaaleaacqWGPbqAaeqaaOGaemiEaG3aaSbaaSqaaiabdMgaPjaaxMaacaWLjaWaaeWaaeaacqaIXaqmaiaawIcacaGLPaaaaeqaaaqaaiabdMgaPjabg2da9iabigdaXaqaaiabd6gaUbqdcqGHris5aaaa@4EEC@

where *x*_*i *_are the spectral features described previously and *c*_*i *_the corresponding coefficients. However, unlike LDA, logistic regression express *D *as a probability of being "true" through the use of a logistic function, such that,

θ=11+e−D(x1,...,xn)     (2)
 MathType@MTEF@5@5@+=feaafiart1ev1aaatCvAUfKttLearuWrP9MDH5MBPbIqV92AaeXatLxBI9gBaebbnrfifHhDYfgasaacH8akY=wiFfYdH8Gipec8Eeeu0xXdbba9frFj0=OqFfea0dXdd9vqai=hGuQ8kuc9pgc9s8qqaq=dirpe0xb9q8qiLsFr0=vr0=vr0dc8meaabaqaciaacaGaaeqabaqabeGadaaakeaaiiGacqWF4oqCcqGH9aqpdaWcaaqaaiabigdaXaqaaiabigdaXiabgUcaRiabdwgaLnaaCaaaleqabaGaeyOeI0IaemiraqKaeiikaGIaemiEaG3aaSbaaWqaaiabigdaXaqabaWccqGGSaalcqGGUaGlcqGGUaGlcqGGUaGlcqGGSaalcqWG4baEdaWgaaadbaGaemOBa4gabeaaliabcMcaPaaaaaGccaWLjaGaaCzcamaabmaabaGaeGOmaidacaGLOaGaayzkaaaaaa@455D@

where *θ *is a value between 0 and 1, indicating the probability of a spectrum with value *D *being "true" or in our case, being an identifiable spectrum. The principle of maximum likelihood is applied by iteratively finding the best estimates for coefficients *c*_*i *_such that the training data best fit equation 2. Unlike LDA, the discriminant function *D *is maximized by maximum likelihood, relaxing the assumptions required to construct the model. For example, the features do not need to be normally distributed and the number of identifiable and unidentifiable training samples need not be similar. Further, because the maximization of the logistic function is probability based, the significance of features can be easily evaluated through analysis of the t-statistic, given the standard errors of the estimated coefficients.

We selected spectral features for the final model in an iterative process, removing features that did not contribute significantly to the final discriminant model. A logistic regression model was computed using all features available, using the computed coefficient and t-statistic for each variable to interpret their contribution to the model. It was found that *IntnRatio20% *and *H*_2_*ORatio *did not contribute significantly based on our training set (see Additional file 2: Appendix 3). These features were then removed and the discriminant model recalculated.

### Statistical modeling of the identifiable and unidentifiable spectra distributions

Once the features of a spectrum are combined into a discriminant score, a statistical model can be used to assess the likelihood of that spectrum being identifiable or unidentifiable based on the spectra distributions of the complete dataset. The first step is to build models for the identifiable and the unidentifiable spectra.

**Figure 1:**
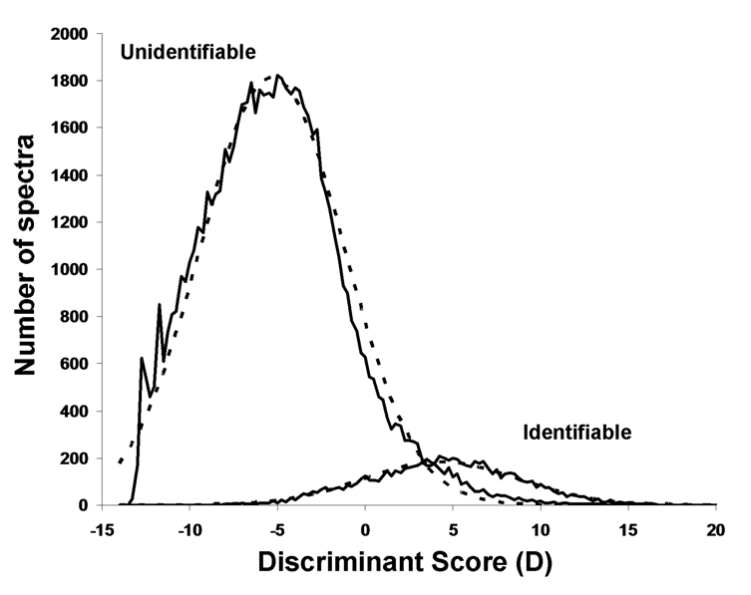
**Modeling identifiable mass spectra using discriminant scoring of spectral features**. The distributions of identifiable and unidentifiable spectra in the UCD test dataset were plotted. The number of spectra is calculated with spectra placed in bins of 0.25 for the discriminant score. The solid lines show the actual distributions of spectra while the dotted lines indicate the estimated Gaussian distributions used to model each distribution.

Figure 1 shows the distribution for the identified and unidentified UCD test spectra tallied within bins of width 0.25 according to the discriminant score, *D*. It is evident that identifiable spectra approximately follow a Gaussian distribution (dotted line), while the distribution of the unidentifiable spectra has a slight positive skew (longer right-tail), probably resulting from high quality but unidentifiable spectra (i.e. misclassified) (see e.g. [11,12]). Thus, it would be reasonable to disregard the skewness and also model the unidentifiable spectra using a Gaussian distribution. Based on Gaussian distributions, the probability that a spectrum has a discriminant score, *D*, given that it is identifiable can be computed as,

p(D|+)=1σ+2πe−(D−μ+)/2σ+ 2     (3)
 MathType@MTEF@5@5@+=feaafiart1ev1aaatCvAUfKttLearuWrP9MDH5MBPbIqV92AaeXatLxBI9gBaebbnrfifHhDYfgasaacH8akY=wiFfYdH8Gipec8Eeeu0xXdbba9frFj0=OqFfea0dXdd9vqai=hGuQ8kuc9pgc9s8qqaq=dirpe0xb9q8qiLsFr0=vr0=vr0dc8meaabaqaciaacaGaaeqabaqabeGadaaakeaacqWGWbaCcqGGOaakcqWGebarcqGG8baFcqGHRaWkcqGGPaqkcqGH9aqpdaWcaaqaaiabigdaXaqaaGGaciab=n8aZnaaBaaaleaacqGHRaWkaeqaaOWaaOaaaeaacqaIYaGmcqWFapaCaSqabaaaaOGaemyzau2aaWbaaSqabeaacqGHsislcqGGOaakcqWGebarcqGHsislcqWF8oqBdaWgaaadbaGaey4kaScabeaaliabcMcaPiabc+caViabikdaYiab=n8aZnaaDaaameaacqqGRaWkaeaacqqGGaaicqqGYaGmaaaaaOGaaCzcaiaaxMaadaqadaqaaiabiodaZaGaayjkaiaawMcaaaaa@4DF7@

where + corresponds to "identifiable spectrum", *μ*_+ _and *σ*_+ _are the mean and standard deviation of the distribution respectively. The conditional probability *p*(*D*|-), where – corresponds to "unidentifiable spectrum" is similarly computed using the mean and standard deviation of the unidentifiable spectra distribution.

Since the estimated distributions will not match the observed distributions for each new dataset, the problem may now be treated as learning a mixture of two Gaussian distributions. An efficient algorithm that is commonly applied to perform unsupervised learning of mixture models is the expectation-maximization (EM) algorithm [24,25]. This algorithm calculates the maximum likelihood estimation for fitting a given model. The PeptideProphet [15] software for predicting likelihoods for a correctly annotated MS/MS spectrum uses a similar approach. In this case, the EM algorithm is initialized using the prior probabilities and parameters of the distributions estimated from our combined test dataset (Figure 1). The EM algorithm optimizes these by iteratively calculating the expected probability assignment, *p*(+|*D*) for each spectrum, using equation 3 and Bayes' Law

p(+|D)=p(D|+)p(+)p(D|+)p(+)+p(D|−)p(−)     (4)
 MathType@MTEF@5@5@+=feaafiart1ev1aaatCvAUfKttLearuWrP9MDH5MBPbIqV92AaeXatLxBI9gBaebbnrfifHhDYfgasaacH8akY=wiFfYdH8Gipec8Eeeu0xXdbba9frFj0=OqFfea0dXdd9vqai=hGuQ8kuc9pgc9s8qqaq=dirpe0xb9q8qiLsFr0=vr0=vr0dc8meaabaqaciaacaGaaeqabaqabeGadaaakeaacqWGWbaCcqGGOaakcqGHRaWkcqGG8baFcqWGebarcqGGPaqkcqGH9aqpdaWcaaqaaiabdchaWjabcIcaOiabdseaejabcYha8jabgUcaRiabcMcaPiabdchaWjabcIcaOiabgUcaRiabcMcaPaqaaiabdchaWjabcIcaOiabdseaejabcYha8jabgUcaRiabcMcaPiabdchaWjabcIcaOiabgUcaRiabcMcaPiabgUcaRiabdchaWjabcIcaOiabdseaejabcYha8jabgkHiTiabcMcaPiabdchaWjabcIcaOiabgkHiTiabcMcaPaaacaWLjaGaaCzcamaabmaabaGaeGinaqdacaGLOaGaayzkaaaaaa@58AC@

In turn the EM algorithm uses the expected probability estimated to optimize the prior probability, *p*(+) and parameters *σ *and *μ *for the + and – distributions, such that

p(+)=1n∑i=1np(+|Di)     (5)
 MathType@MTEF@5@5@+=feaafiart1ev1aaatCvAUfKttLearuWrP9MDH5MBPbIqV92AaeXatLxBI9gBaebbnrfifHhDYfgasaacH8akY=wiFfYdH8Gipec8Eeeu0xXdbba9frFj0=OqFfea0dXdd9vqai=hGuQ8kuc9pgc9s8qqaq=dirpe0xb9q8qiLsFr0=vr0=vr0dc8meaabaqaciaacaGaaeqabaqabeGadaaakeaacqWGWbaCcqGGOaakcqGHRaWkcqGGPaqkcqGH9aqpdaWcaaqaaiabigdaXaqaaiabd6gaUbaadaaeWbqaaiabdchaWjabcIcaOiabgUcaRiabcYha8jabdseaenaaBaaaleaacqWGPbqAaeqaaOGaeiykaKcaleaacqWGPbqAcqGH9aqpcqaIXaqmaeaacqWGUbGBa0GaeyyeIuoakiaaxMaacaWLjaWaaeWaaeaacqaI1aqnaiaawIcacaGLPaaaaaa@46FA@

where *n *is the number of spectra in the dataset.

μ+=1p(+)n∑i=1np(+|Di)Di     (6)
 MathType@MTEF@5@5@+=feaafiart1ev1aaatCvAUfKttLearuWrP9MDH5MBPbIqV92AaeXatLxBI9gBaebbnrfifHhDYfgasaacH8akY=wiFfYdH8Gipec8Eeeu0xXdbba9frFj0=OqFfea0dXdd9vqai=hGuQ8kuc9pgc9s8qqaq=dirpe0xb9q8qiLsFr0=vr0=vr0dc8meaabaqaciaacaGaaeqabaqabeGadaaakeaaiiGacqWF8oqBdaWgaaWcbaGaey4kaScabeaakiabg2da9maalaaabaGaeGymaedabaGaemiCaaNaeiikaGIaey4kaSIaeiykaKIaemOBa4gaamaaqahabaGaemiCaaNaeiikaGIaey4kaSIaeiiFaWNaemiraq0aaSbaaSqaaiabdMgaPbqabaGccqGGPaqkcqWGebardaWgaaWcbaGaemyAaKgabeaakiaaxMaacaWLjaWaaeWaaeaacqaI2aGnaiaawIcacaGLPaaaaSqaaiabdMgaPjabg2da9iabigdaXaqaaiabd6gaUbqdcqGHris5aaaa@4C69@

σ+=1p(+)n∑i=1np(+|Di)(Di−μ)2     (7)
 MathType@MTEF@5@5@+=feaafiart1ev1aaatCvAUfKttLearuWrP9MDH5MBPbIqV92AaeXatLxBI9gBaebbnrfifHhDYfgasaacH8akY=wiFfYdH8Gipec8Eeeu0xXdbba9frFj0=OqFfea0dXdd9vqai=hGuQ8kuc9pgc9s8qqaq=dirpe0xb9q8qiLsFr0=vr0=vr0dc8meaabaqaciaacaGaaeqabaqabeGadaaakeaaiiGacqWFdpWCdaWgaaWcbaGaey4kaScabeaakiabg2da9maakaaabaWaaSaaaeaacqaIXaqmaeaacqWGWbaCcqGGOaakcqGHRaWkcqGGPaqkcqWGUbGBaaWaaabCaeaacqWGWbaCcqGGOaakcqGHRaWkcqGG8baFcqWGebardaWgaaWcbaGaemyAaKgabeaakiabcMcaPiabcIcaOiabdseaenaaBaaaleaacqWGPbqAaeqaaOGaeyOeI0Iae8hVd0MaeiykaKYaaWbaaSqabeaacqaIYaGmaaaabaGaemyAaKMaeyypa0JaeGymaedabaGaemOBa4ganiabggHiLdaaleqaaOGaaCzcaiaaxMaadaqadaqaaiabiEda3aGaayjkaiaawMcaaaaa@5201@

The prior probability for the unidentifiable spectra is (1-*p*(+)). The distribution parameters are calculated similarly but use (1-*p*(+|*D*_*i*_)) as the likelihood estimate for a spectrum being unidentifiable.

The EM algorithm is allowed to run until there are no significant changes to the estimated parameters between iterations. Supplementary Figure 2 (see Additional file 3) show examples of the algorithm used to fit datasets with different spectra distributions. In general the predicted identifiable and unidentifiable spectra distributions match the observed well, especially for the UCD dataset. For the ISB example, the unidentified spectra distribution is less well modeled; this may be a consequence of the smaller number of spectra available, or may reflect the need to include an additional distribution for effective modeling. Nevertheless, the predicted model still provides a reasonable estimate and importantly, the identifiable distribution is modeled well which is of principal importance for finding high quality spectra.

**Figure 2:**
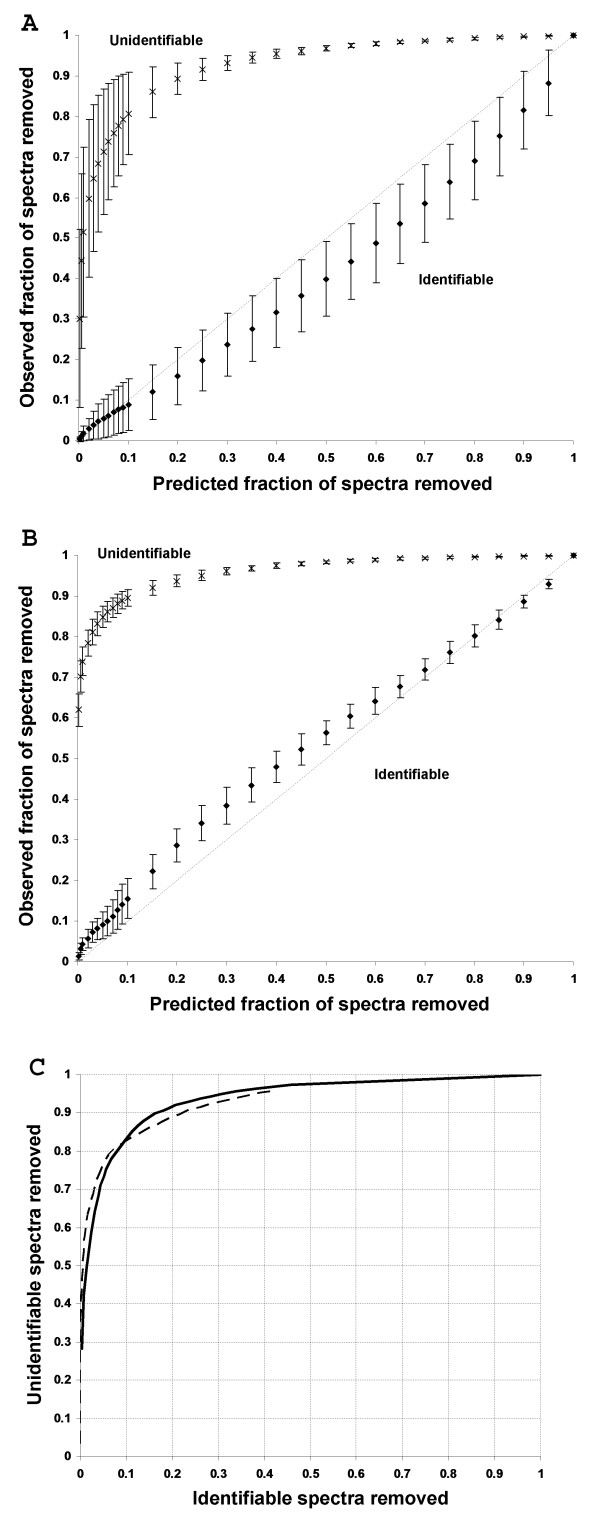
**Removal unidentifiable spectra by msmsEval**. The predicted fraction of spectra removed for identifiable (◆) and unidentifiable (**x**) spectra were plotted against the observed fractions for 10 runs of the UCD test dataset (A) and 22 runs of the ISB test dataset (B). The estimated fraction of spectra removed is calculated by taking the respective percentiles from the identifiable spectra Gaussian distributions. The diagonal thin dashed line shows expected trend for the removal of identifiable spectra if the estimated values match the observed values perfectly. Error bars are one standard deviation from the average of the respective test datasets. Receiver operator curves showing the fraction of identifiable spectra removed versus unidentifiable spectra removed for the UCD test dataset (solid line) and ISB dataset (dashed line) are also shown (C).

### Illustrative examples

#### Removal of low quality unidentifiable spectra

We demonstrate the use of the assigned probabilities as a method for removing spectra that are unlikely to be identifiable from the test datasets. Runs of the UCD test dataset and ISB dataset were each analyzed and modeled separately using msmsEval. To demonstrate the accuracy of the algorithm, the estimated fraction of identifiable spectra removed at various points (the estimated fraction is calculated from the corresponding percentile values from the identifiable spectra Gaussian plot) is plotted against the actual observed fractions (Figure 2A&B).

For the UCD test dataset (Figure 2A), it can be seen that observed and predicted fractions for the identified spectra show reasonable agreement. The error bars indicate that there is some variance in the prediction between the datasets, this is likely due to the diversity of runs within the test dataset. As expected, the variance between runs from the ISB dataset is lower than for the UCD dataset (Figure 2B). This is because runs within the ISB dataset contain a low number of proteins, are of similar constituents and was presumably acquired during a single study. In the UCD case the data comprised of diverse real world examples from an active proteomics lab. It is also observed that the predicted fraction slightly underestimates the observed fraction, probably because spectra annotated by SEQUEST were not filtered by PeptideProphet (which was used to annotate the ISB dataset) and as a result there are a higher number of low quality identified spectra. Nevertheless, in both cases, the fraction of unidentified spectra removed is significantly higher than identified for fractions less than one.

Figure 2C shows the average fraction of unidentifiable spectra removed in relation to identified. For both datasets the shape of the receiver-operator-curve are similar. In general, the greater the number of unidentified spectra removed, greater the number of identifiable spectra also removed. For practical purposes in a proteomics laboratory, a user will want to maximize the removal of unidentifiable spectra without significant lost of identifiable spectra. From Figure 2C, it can be observed that the removal of 50% of unidentifiable spectra, resulting in a two-fold decrease in computer search time, will remove only 1–2% of identifiable spectra. This is consistent with other reports.

#### Guide for finding modified spectra

**Figure 3:**
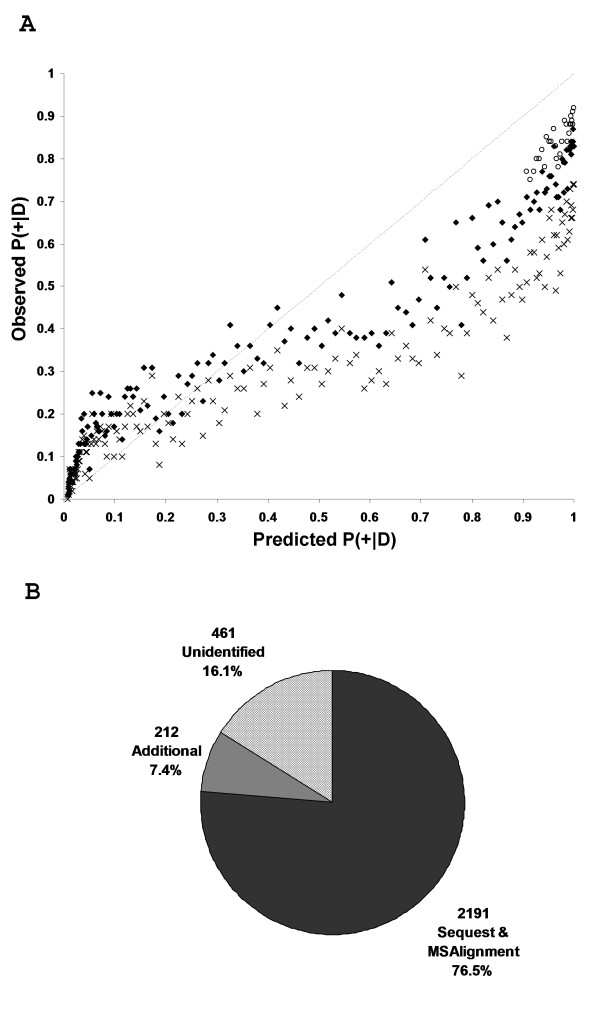
**msmsEval highlights strong candidates for modified peptide spectra**. The observed p(+|*D*) versus predicted p(+|*D*) values for 22 runs of the ISB dataset (A) were plotted using binned sets of 100 spectra (i.e. the fraction of the 100 spectra that were observed to be identifiable versus the mean p(+|*D*)). Observed p(+|*D*) values calculated using SEQUEST identifications (**x**), SEQUEST and MSAlignment/InsPecT identifications (**◆**), and SEQUEST and MSAlignment/InsPecT identifications as well as the additional assignments described in the text (**o**), are indicated. A pie chart (B) shows the absolute numbers and percentages of spectra from the ISB dataset with predicted p(+|*D*) > 0.9 that were identified by SEQUEST/MSAlignment/InsPecT, those that were additionally identified by msmsEval, and those that remain unidentified. In total, 83.9% of spectra with p(+|*D*) > 0.9 were identified.

To demonstrate that the model is reliable in predicting the probability that a spectrum is identifiable, p(+|*D*), the estimated probability is plotted against the observed probability for the ISB dataset (Figure 3A). Spectra were sorted based on the predicted p(+|*D*), and for bins of 100 spectra, the average p(+|*D*) was calculated and plotted against the fraction of observed identifiable spectra in those same bin. Figure 3A shows that when p(+|*D*) is plotted against spectra that were only annotated using SEQUEST (crosses), the observed probabilities underestimate the predicted probabilities (e.g. only 60% of spectra with predicted p(+|*D*) = 0.9 are being identified when one would expect 90%). However with the addition of the spectra representing mostly modified peptide annotated by Tsur and coworkers (2005) [18], it can be seen that the observed probability becomes significantly closer to the predicted probability (diamonds).

Despite the improvement, the estimated probability still appears to over-estimate the observed, particularly for higher p(+|*D*). To investigate whether this discrepancy all remaining unidentified spectra with p(+|*D*) > 0.9 (673 spectra) were further analysed as described in the experimental section. Of these spectra, 212 were deemed to be correctly annotated confirming that there were indeed unannotated high quality spectra that were still unidentified. The newly annotated spectra belonged to one of the following three categories (a full list with annotation is available at the msmsEval website):

1) Two new proteins. A number of spectra were annotated as peptides of bovine alpha-S1 and alpha-S2-casein which are proteins not from the original set of 18 standard proteins. 68 and 61 spectra were assigned to unique tryptic fragments of one of these proteins by SEQUEST and InsPecT respectively against a UniProt database (see experimental section for search description) giving very strong evidence that the annotations are correct. The bovine beta-casein preparation from Sigma C6905 is only > 90% pure, while alpha casein is also present in bovine milk.

2) Further spectra of unmodified peptides of one of the 18 standard proteins were annotated. These were found by increasing the tolerance for mass errors allowed in our searches.

3) Spectra of modified peptides of one of the 18 standard proteins. It is perhaps surprising that previous efforts by Tsur and coworkers [18] did not identify these spectra, however this could be due to a number of factors including the use of different parameters (e.g. maximum number of modifications allowed) or a different version of InsPecT. The majority of modifications observed were multiple methionine oxidations. However, we also observed amino acid polymorphisms. For example the mutation of Gln-1871 into Pro in rabbit myosin heavy chain. There is sufficient evidence that this annotation is correct as it is observed 10 times in the InsPecT blind search, in all cases with a p-value below 0.05. In addition, the mutation of glutamine into proline is reasonable given that it only requires the mutation of a single DNA base.

Notably, the inclusion of the newly annotated spectra into Figure 3A increases the correspondence between the predicted p(+|*D*) and the observed probability (circles). In fact, 83.7% of all spectra in the ISB dataset with a predicted p(+|*D*) of greater than 0.9 have now been annotated (Figure 3B).

## Discussion

Our results and previous work show that the ability to assess the quality of tandem mass spectra independent of database searching can improve throughput and performance of MS-based proteomics projects. We demonstrated the use of msmsEval on data from different real life sources, and show that computed probabilities reflect the observed values, and are therefore useful for removing low quality spectra prior to database searching, and/or for locating high quality unassigned spectra. For instance, removing 50% of poor quality (unidentified) spectra would result in removal of only ~2% (UCD) to ~1% (ISB) of high quality (identifiable spectra). Notably, our algorithm automatically adjusts the p(+|*D*) values to the slight variations of spectral quality distributions in the different datasets.

Ideally, the spectral quality discriminant function should be retrained for each type of data acquired on different instruments [12]. The EM algorithm automatically adapts the statistical models to different discriminant functions and will account for variations between datasets. Nevertheless, the use of a new discriminant function specific to each dataset should provide greater accuracy, and our software implementation, msmsEval, provides the ability to use newly trained discriminant functions. Furthermore, while generally the EM algorithm will converge on the correct local minimum given a large sample size (i.e. more than 500 spectra), it is acknowledge that skewed datasets such as those with very few identifiable spectra will pose a greater challenge for the EM algorithm to converge at the correct solution. To increase the robustness of the EM algorithm, options are available in the software to constrain the Gaussian mixture model parameters. Like the discriminant function, such constrains can be altered based on new training datasets.

While the major contribution of this work is the development of a method to statistically model the quality distribution of tandem mass spectra, we have also shown that there are indeed significant numbers of high quality spectra that remain unannotated in multidimensional LC/MS/MS experiments and that these can be retrieved by msmsEval with high confidence. Using the well annotated ISB dataset, we show that this may reflect the presence of unknown contaminants, amino acid polymorphisms, or post-translational modifications. Currently attempts are being made to annotate high quality unidentified samples from our UCD dataset. As many of the samples are from "real life" human vascular proteins, it is anticipated that the range of unknown proteins, posttranslational modifications and amino acid polymorphisms will be significantly greater than those present in standard datasets such as the ISB dataset. Efforts to annotate such spectra will require the use of translated genome sequences in combination with blind searches for unanticipated modifications. These analyses are extremely time consuming (more than a minute per megabyte of database searched using InsPecT in blind mode per spectrum) and the use of spectra quality assessment is a major step towards enabling such searches.

## Conclusion

A probability based method of assessing the quality of peptide tandem mass spectra has been developed by empirically modeling the distribution of spectra within a single or multidimensional LC/MS/MS run based on selected spectral features. The ability to rapidly and accurately estimate the quality of a tandem mass spectrum brings significant benefits to high-throughput proteomics, 1) the fraction of potentially identifiable spectra that may be removed can be estimated when rejecting low quality spectra, and 2) the probability that a given spectrum will be identifiable will allow greater selectivity when focusing efforts toward identifying unannotated high quality spectra.

## Methods

### Generation and annotation of experimental dataset

All spectra were acquired from ion trap mass spectrometers with electrospray ion sources following LC, LC-LC or MudPIT type experiments. Cysteine thiol groups were reduced and alkylated using iodoacetamide. Subsequently samples were digested with trypsin and the resulting mixtures were separated using reverse phase and cation exchange chromatography prior to mass spectrometry analysis as described in Cagney *et *al. [26]. Three different database search strategies were used to annotate the dataset.

**a**. SEQUEST/PeptideProphet/ProteinProphet. Spectra were first searched using SEQUEST, with all multiply charged spectra search twice as 2+ and 3+ against the SwissProt database (UniProtKB/Swiss-Prot, Release 6.0) Carboxymethylation of cysteines (+57) was specified as a fixed modification while methionine oxidation (+18) was specified as a variable modification. Subsequently, the results were evaluated using PeptideProphet [15] and ProteinProphet [16]. All spectra with a protein and peptide probability of greater than 0.9 were deemed as correctly identified.

**b**. InsPecT. Spectra were searched using the InsPecT [17], a tag-based database search algorithm. The same amino acid modifications were specified as for the SEQUEST search. To determine whether a spectrum was correctly annotated, a list of candidate proteins that are supported by two or more unique peptides was first generated. Subsequently, all spectra within the dataset that have a p-value of < 0.05 and are annotated as a peptide of a protein from the list of candidate proteins were deemed as correctly identified.

**c**. pepNovo/SPIDER. pepNovo [27] was used to infer an amino acid sequence for each spectrum. In addition, up to ten sequence tags of lengths 4, 5 and 6 were also generated. For each spectrum, the inferred amino acid sequence and the sets of tags were used as input for SPIDER [19], such that for each spectrum, four SPIDER searches, one for each set of tag lengths and one for the full sequence, were performed. The candidate protein with the maximal combined score was temporarily assigned to each spectrum. Subsequently, the same procedure used for InsPecT was applied to determine whether a spectrum has been correctly annotated.

### Spectral feature definition

1. *NPeaks*. The number of peaks in a spectrum.

2. *NormTIC*. Normalized total ion count, where the value is the ratio of the total ion count of the current spectrum to the mean total ion count of all spectra within the same run.

NormTIC=TICMeanTICMeanTIC=1n∑i=1nTICi     (1)
 MathType@MTEF@5@5@+=feaafiart1ev1aaatCvAUfKttLearuWrP9MDH5MBPbIqV92AaeXatLxBI9gBaebbnrfifHhDYfgasaacH8akY=wiFfYdH8Gipec8Eeeu0xXdbba9frFj0=OqFfea0dXdd9vqai=hGuQ8kuc9pgc9s8qqaq=dirpe0xb9q8qiLsFr0=vr0=vr0dc8meaabaqaciaacaGaaeqabaqabeGadaaakeaafaqaaeGadaaabaGaemOta4Kaem4Ba8MaemOCaiNaemyBa0MaemivaqLaemysaKKaem4qameabaGaeyypa0dabaWaaSaaaeaacqWGubavcqWGjbqscqWGdbWqaeaacqWGnbqtcqWGLbqzcqWGHbqycqWGUbGBcqWGubavcqWGjbqscqWGdbWqaaaabaGaemyta0KaemyzauMaemyyaeMaemOBa4MaemivaqLaemysaKKaem4qameabaGaeyypa0dabaWaaSaaaeaacqaIXaqmaeaacqWGUbGBaaWaaabCaeaacqWGubavcqWGjbqscqWGdbWqdaWgaaWcbaGaemyAaKgabeaaaeaacqWGPbqAcqGH9aqpcqaIXaqmaeaacqWGUbGBa0GaeyyeIuoaaaGccaWLjaGaaCzcamaabmaabaGaeGymaedacaGLOaGaayzkaaaaaa@5DE9@

3. *GoodSegs*. The ratio of the number of segments, *i*, of size 56 m/z that are likely to be occupied by a *b *or *y *ion, to the total number of occupied and unoccupied segments such that,

GoodSegs=∑segi∑i i∈{max⁡(mz)56}segi={1ifIntn(xrank(1))>Intn(xrank(5))×30else} x∈i     (2)
 MathType@MTEF@5@5@+=feaafiart1ev1aaatCvAUfKttLearuWrP9MDH5MBPbIqV92AaeXatLxBI9gBaebbnrfifHhDYfgasaacH8akY=wiFfYdH8Gipec8Eeeu0xXdbba9frFj0=OqFfea0dXdd9vqai=hGuQ8kuc9pgc9s8qqaq=dirpe0xb9q8qiLsFr0=vr0=vr0dc8meaabaqaciaacaGaaeqabaqabeGadaaakeaafaqaaeGabaaabaGaem4raCKaem4Ba8Maem4Ba8MaemizaqMaem4uamLaemyzauMaem4zaCMaem4CamNaeyypa0ZaaSaaaeaadaaeabqaaiabdohaZjabdwgaLjabdEgaNnaaBaaaleaacqWGPbqAaeqaaaqabeqaniabggHiLdaakeaadaaeabqaaiabdMgaPbWcbeqab0GaeyyeIuoaaaGccqqGGaaicqWGPbqAcqGHiiIZdaGadeqaamaalaaabaGagiyBa0MaeiyyaeMaeiiEaGNaeiikaGIaemyBa0MaemOEaONaeiykaKcabaGaeGynauJaeGOnaydaaaGaay5Eaiaaw2haaaqaaiabdohaZjabdwgaLjabdEgaNnaaBaaaleaacqWGPbqAaeqaaOGaeyypa0ZaaiWabeaafaqaaeGadaaabaGaeGymaedabaGaemyAaKMaemOzaygabaGaemysaKKaemOBa4MaemiDaqNaemOBa4MaeiikaGIaemiEaG3aaSbaaSqaaiabdkhaYjabdggaHjabd6gaUjabdUgaRjabcIcaOiabigdaXiabcMcaPaqabaGccqGGPaqkcqGH+aGpcqWGjbqscqWGUbGBcqWG0baDcqWGUbGBcqGGOaakcqWG4baEdaWgaaWcbaGaemOCaiNaemyyaeMaemOBa4Maem4AaSMaeiikaGIaeGynauJaeiykaKcabeaakiabcMcaPiabgEna0kabiodaZaqaaiabicdaWaqaaiabdwgaLjabdYgaSjabdohaZjabdwgaLbqaaaaaaiaawUhacaGL9baacqqGGaaicqWG4baEcqGHiiIZcqWGPbqAaaGaaCzcaiaaxMaadaqadaqaaiabikdaYaGaayjkaiaawMcaaaaa@95A5@

where *Intn*(*x*_*rank*(*1*)_) and *Intn*(*x*_*rank*(*5*)_) are the intensity of the first and fifth most intense peaks (*x*_*rank*(*1*), _*x*_*rank*(*5*)_) within segment, *i*, respectively. Likely *b *or *y *ion occupation of a segment is assessed by comparing the most and fifth-most intense peaks to reduce the possibility of comparing *b *and *y *ions that exist within the same segment, or that represent isotope shoulder peaks. If there are fewer than five peaks within *i*, then the least intense peak is used.

4. *IntnRatio1%*. The ratio of peaks that have a relative intensity of greater than 1% of total intensity

IntnRatio=∑ini∈{relativeIntensity>1%}     (3)
 MathType@MTEF@5@5@+=feaafiart1ev1aaatCvAUfKttLearuWrP9MDH5MBPbIqV92AaeXatLxBI9gBaebbnrfifHhDYfgasaacH8akY=wiFfYdH8Gipec8Eeeu0xXdbba9frFj0=OqFfea0dXdd9vqai=hGuQ8kuc9pgc9s8qqaq=dirpe0xb9q8qiLsFr0=vr0=vr0dc8meaabaqaciaacaGaaeqabaqabeGadaaakeaafaqaaeqacaaabaGaemysaKKaemOBa4MaemiDaqNaemOBa4MaemOuaiLaemyyaeMaemiDaqNaemyAaKMaem4Ba8Maeyypa0ZaaSaaaeaadaaeabqaaiabdMgaPbWcbeqab0GaeyyeIuoaaOqaaiabd6gaUbaaaeaacqWGPbqAcqGHiiIZcqGG7bWEcqWGYbGCcqWGLbqzcqWGSbaBcqWGHbqycqWG0baDcqWGPbqAcqWG2bGDcqWGLbqzcqWGjbqscqWGUbGBcqWG0baDcqWGLbqzcqWGUbGBcqWGZbWCcqWGPbqAcqWG0baDcqWG5bqEcqGH+aGpcqaIXaqmcqGGLaqjcqGG9bqFcaWLjaGaaCzcamaabmaabaGaeG4mamdacaGLOaGaayzkaaaaaaaa@6276@

5. *IntnRatio20%*. The ratio of peaks that has a relative intensity of greater than 20% of total intensity

IntnRatio=∑ini∈{relativeIntensity>20%}     (4)
 MathType@MTEF@5@5@+=feaafiart1ev1aaatCvAUfKttLearuWrP9MDH5MBPbIqV92AaeXatLxBI9gBaebbnrfifHhDYfgasaacH8akY=wiFfYdH8Gipec8Eeeu0xXdbba9frFj0=OqFfea0dXdd9vqai=hGuQ8kuc9pgc9s8qqaq=dirpe0xb9q8qiLsFr0=vr0=vr0dc8meaabaqaciaacaGaaeqabaqabeGadaaakeaafaqaaeqacaaabaGaemysaKKaemOBa4MaemiDaqNaemOBa4MaemOuaiLaemyyaeMaemiDaqNaemyAaKMaem4Ba8Maeyypa0ZaaSaaaeaadaaeabqaaiabdMgaPbWcbeqab0GaeyyeIuoaaOqaaiabd6gaUbaaaeaacqWGPbqAcqGHiiIZcqGG7bWEcqWGYbGCcqWGLbqzcqWGSbaBcqWGHbqycqWG0baDcqWGPbqAcqWG2bGDcqWGLbqzcqWGjbqscqWGUbGBcqWG0baDcqWGLbqzcqWGUbGBcqWGZbWCcqWGPbqAcqWG0baDcqWG5bqEcqGH+aGpcqaIYaGmcqaIWaamcqGGLaqjcqGG9bqFcaWLjaGaaCzcamaabmaabaGaeGinaqdacaGLOaGaayzkaaaaaaaa@6368@

Note that measuring the intensity ratio cut-offs (equation 3 and 4) may appear to have statistical independence issues. However, as there is no relationship between the number of peaks between 0–1% and 1–20% of total intensity (data not shown; [11]), the result of equations 3 & 4 should be independent.

6. *Complements*. Pairs of peaks whose m/z values add together to give the m/z of the parent. The feature is computed for the most commonly observed charge states of 1+, 2+ or 3+. The charge state with the greatest feature value is used. The feature value is calculated as follows:

Complements=∑i1max⁡(rank(x),rank(y))i∈{mz(x)+mz(y)≈Mparent}     (5)
 MathType@MTEF@5@5@+=feaafiart1ev1aaatCvAUfKttLearuWrP9MDH5MBPbIqV92AaeXatLxBI9gBaebbnrfifHhDYfgasaacH8akY=wiFfYdH8Gipec8Eeeu0xXdbba9frFj0=OqFfea0dXdd9vqai=hGuQ8kuc9pgc9s8qqaq=dirpe0xb9q8qiLsFr0=vr0=vr0dc8meaabaqaciaacaGaaeqabaqabeGadaaakeaafaqaaeqacaaabaGaem4qamKaem4Ba8MaemyBa0MaemiCaaNaemiBaWMaemyzauMaemyBa0MaemyzauMaemOBa4MaemiDaqNaem4CamNaeyypa0ZaaabuaeaadaWcaaqaaiabigdaXaqaaiGbc2gaTjabcggaHjabcIha4jabcIcaOiabdkhaYjabdggaHjabd6gaUjabdUgaRjabcIcaOiabdIha4jabcMcaPiabcYcaSiabdkhaYjabdggaHjabd6gaUjabdUgaRjabcIcaOiabdMha5jabcMcaPiabcMcaPaaaaSqaaiabdMgaPbqab0GaeyyeIuoaaOqaaiabdMgaPjabgIGiolabcUha7jabd2gaTjabdQha6jabcIcaOiabdIha4jabcMcaPiabgUcaRiabd2gaTjabdQha6jabcIcaOiabdMha5jabcMcaPiabgIKi7kabd2eannaaBaaaleaacqWGWbaCcqWGHbqycqWGYbGCcqWGLbqzcqWGUbGBcqWG0baDaeqaaOGaeiyFa0NaaCzcaiaaxMaadaqadaqaaiabiwda1aGaayjkaiaawMcaaaaaaaa@7B31@

An error margin of ± 1 was used for parent mass matching. For peptides with charge 2+ or more, the formula is adjusted appropriately, i.e. for 3+, one of the fragment ions is multiplied by 2. This implements a method similar to 2 to 3 [28] where the aim is to estimate the charge state of a precursor peptide by observing complementary peaks.

7. *IsoRatio*. The presence of isotope peaks associated with an inferred *b *or *y *ion by measuring the proportion of peaks of rank 1 or 2 associated with an isotope peak, such that,

IsoRatio=∑isoi∑i i∈{max⁡(mz)56}isoi={1ifxy−xrank(k)≈1y∈all,k∈{1,2}0else} x∈i     (6)
 MathType@MTEF@5@5@+=feaafiart1ev1aaatCvAUfKttLearuWrP9MDH5MBPbIqV92AaeXatLxBI9gBaebbnrfifHhDYfgasaacH8akY=wiFfYdH8Gipec8Eeeu0xXdbba9frFj0=OqFfea0dXdd9vqai=hGuQ8kuc9pgc9s8qqaq=dirpe0xb9q8qiLsFr0=vr0=vr0dc8meaabaqaciaacaGaaeqabaqabeGadaaakeaafaqaaeGabaaabaGaemysaKKaem4CamNaem4Ba8MaemOuaiLaemyyaeMaemiDaqNaemyAaKMaem4Ba8Maeyypa0ZaaSaaaeaadaaeabqaaiabdMgaPjabdohaZjabd+gaVnaaBaaaleaacqWGPbqAaeqaaaqabeqaniabggHiLdaakeaadaaeabqaaiabdMgaPbWcbeqab0GaeyyeIuoaaaGccqqGGaaicqWGPbqAcqGHiiIZdaGadeqaamaalaaabaGagiyBa0MaeiyyaeMaeiiEaGNaeiikaGIaemyBa0MaemOEaONaeiykaKcabaGaeGynauJaeGOnaydaaaGaay5Eaiaaw2haaaqaaiabdMgaPjabdohaZjabd+gaVnaaBaaaleaacqWGPbqAaeqaaOGaeyypa0ZaaiWabeaafaqaaeGaeaaaaeaacqaIXaqmaeaacqWGPbqAcqWGMbGzaeaacqWG4baEdaWgaaWcbaGaemyEaKhabeaakiabgkHiTiabdIha4naaBaaaleaacqWGYbGCcqWGHbqycqWGUbGBcqWGRbWAcqGGOaakcqWGRbWAcqGGPaqkaeqaaOGaeyisISRaeGymaedabaacbiGae8xEaKNaeyicI4Sae8xyaeMae8hBaWMae8hBaWMae8hlaWIae83AaSMaeyicI48aaiWabeaacqaIXaqmcqGGSaalcqaIYaGmaiaawUhacaGL9baaaeaacqaIWaamaeaacqWGLbqzcqWGSbaBcqWGZbWCcqWGLbqzaeaaaeaaaaaacaGL7bGaayzFaaGaeeiiaaIaemiEaGNaeyicI4SaemyAaKgaaiaaxMaacaWLjaWaaeWaaeaacqaI2aGnaiaawIcacaGLPaaaaaa@90E4@

An error margin of ± 0.3 was used for parent mass matching.

8. *H*_2_*ORatio*. The presence of water loss peaks associated with inferred *b *or *y *ion by measuring the proportion of peaks ranked 1 or 2 associated with a water loss, such that,

H2ORatio=∑H2Oi∑i i∈{max⁡(mz)56}H2Oi={1ifxrank(k)−xy≈18y∈all,k∈{1,2}0else} x∈i     (7)
 MathType@MTEF@5@5@+=feaafiart1ev1aaatCvAUfKttLearuWrP9MDH5MBPbIqV92AaeXatLxBI9gBaebbnrfifHhDYfgasaacH8akY=wiFfYdH8Gipec8Eeeu0xXdbba9frFj0=OqFfea0dXdd9vqai=hGuQ8kuc9pgc9s8qqaq=dirpe0xb9q8qiLsFr0=vr0=vr0dc8meaabaqaciaacaGaaeqabaqabeGadaaakeaafaqaaeGabaaabaGaemisaG0aaSbaaSqaaiabikdaYaqabaGccqWGpbWtcqWGsbGucqWGHbqycqWG0baDcqWGPbqAcqWGVbWBcqGH9aqpdaWcaaqaamaaqaeabaGaemisaG0aaSbaaSqaaiabikdaYaqabaGccqWGpbWtdaWgaaWcbaGaemyAaKgabeaaaeqabeqdcqGHris5aaGcbaWaaabqaeaacqWGPbqAaSqabeqaniabggHiLdaaaOGaeeiiaaIaemyAaKMaeyicI48aaiWabeaadaWcaaqaaiGbc2gaTjabcggaHjabcIha4jabcIcaOiabd2gaTjabdQha6jabcMcaPaqaaiabiwda1iabiAda2aaaaiaawUhacaGL9baaaeaacqWGibasdaWgaaWcbaGaeGOmaidabeaakiabd+eapnaaBaaaleaacqWGPbqAaeqaaOGaeyypa0ZaaiWabeaafaqaaeGaeaaaaeaacqaIXaqmaeaacqWGPbqAcqWGMbGzaeaacqWG4baEdaWgaaWcbaGaemOCaiNaemyyaeMaemOBa4Maem4AaSMaeiikaGIaem4AaSMaeiykaKcabeaakiabgkHiTiabdIha4naaBaaaleaacqWG5bqEaeqaaOGaeyisISRaeGymaeJaeGioaGdabaacbiGae8xEaKNaeyicI4Sae8xyaeMae8hBaWMae8hBaWMae8hlaWIae83AaSMaeyicI48aaiWabeaacqaIXaqmcqGGSaalcqaIYaGmaiaawUhacaGL9baaaeaacqaIWaamaeaacqWGLbqzcqWGSbaBcqWGZbWCcqWGLbqzaeaaaeaaaaaacaGL7bGaayzFaaGaeeiiaaIaemiEaGNaeyicI4SaemyAaKgaaiaaxMaacaWLjaWaaeWaaeaacqaI3aWnaiaawIcacaGLPaaaaaa@8FC9@

An error margin of ± 0.3 was used for parent mass matching.

9. *AAdiffRatio*. This feature quantifies evidence for *b *or *y *ion pairs separated by amino acid masses by measuring the ratio of the most intense peak within each segment, *i*, that has an associated peak (not necessarily in segment *i*) whose distance from the most intense peak is equivalent to the mass of one of the amino acids ± 0.3, such that,

AAdiffRatio=∑AAdiffj∑j j∈{rank(1)}AAdiffj={1if|xj−xrank(k)|∈{M(AA)±0.3}k∈{1,2}0else} x∈{0,max⁡(mz)}     (8)
 MathType@MTEF@5@5@+=feaafiart1ev1aaatCvAUfKttLearuWrP9MDH5MBPbIqV92AaeXatLxBI9gBaebbnrfifHhDYfgasaacH8akY=wiFfYdH8Gipec8Eeeu0xXdbba9frFj0=OqFfea0dXdd9vqai=hGuQ8kuc9pgc9s8qqaq=dirpe0xb9q8qiLsFr0=vr0=vr0dc8meaabaqaciaacaGaaeqabaqabeGadaaakeaafaqaaeGabaaabaGaemyqaeKaemyqaeKaemizaqMaemyAaKMaemOzayMaemOzayMaemOuaiLaemyyaeMaemiDaqNaemyAaKMaem4Ba8Maeyypa0ZaaSaaaeaadaaeabqaaiabdgeabjabdgeabjabdsgaKjabdMgaPjabdAgaMjabdAgaMnaaBaaaleaacqWGQbGAaeqaaaqabeqaniabggHiLdaakeaadaaeabqaaiabdQgaQbWcbeqab0GaeyyeIuoaaaGccqqGGaaiieGacqWFQbGAcqGHiiIZdaGadeqaaiabdkhaYjabdggaHjabd6gaUjabdUgaRjabcIcaOiabigdaXiabcMcaPaGaay5Eaiaaw2haaaqaaiabdgeabjabdgeabjabdsgaKjabdMgaPjabdAgaMjabdAgaMnaaBaaaleaacqWGQbGAaeqaaOGaeyypa0ZaaiWabeaafaqaaeGaeaaaaeaacqaIXaqmaeaacqWGPbqAcqWGMbGzaeaacqGG8baFcqWG4baEdaWgaaWcbaGaemOAaOgabeaakiabgkHiTiabdIha4naaBaaaleaacqWGYbGCcqWGHbqycqWGUbGBcqWGRbWAcqGGOaakcqWGRbWAcqGGPaqkaeqaaOGaeiiFaWNaeyicI48aaiWabeaacqWGnbqtcqGGOaakcqWGbbqqcqWGbbqqcqGGPaqkcqGHXcqScqaIWaamcqGGUaGlcqaIZaWmaiaawUhacaGL9baaaeaacqWFRbWAcqGHiiIZdaGadeqaaiabigdaXiabcYcaSiabikdaYaGaay5Eaiaaw2haaaqaaiabicdaWaqaaiabdwgaLjabdYgaSjabdohaZjabdwgaLbqaaaqaaaaaaiaawUhacaGL9baacqqGGaaicqWG4baEcqGHiiIZdaGadeqaaiabicdaWiabcYcaSiGbc2gaTjabcggaHjabcIha4jabcIcaOiabd2gaTjabdQha6jabcMcaPaGaay5Eaiaaw2haaaaacaWLjaGaaCzcamaabmaabaGaeGioaGdacaGLOaGaayzkaaaaaa@A95B@

where *M*(*AA*) is the mass of one of the 20 amino acids. Note that modified proteins can be included in the algorithm by substituting non-standard amino acid masses into *AAdiff*, for instance the mass of caboxymethyl-cysteine.

While some of our variables (equations 2, 3, 4, 6, 7) have bounds of 0 to 1, the remaining variables are not bounded. However, through all our observed training and test spectra, no spurious variation were observed and the distribution were smooth and continuous (data not shown) and from our knowledge of the nature of tandem mass spectra in relation to our features, it is a reasonable assumption that this observation may be generalized.

### Further analysis of high quality unidentified spectra

The existence of high quality unannotated spectra within the ISB dataset as predicted by our method prompted further investigation. Three different searches were performed as described below:

1) SEQUEST was performed against the SwissProt database (UniProtKB/Swiss-Prot, Release 6.0). The parent mass tolerance was set at +/- 5. Furthermore, methionine oxidation and cysteine caboxylmethylation were specified as optional.

2) InsPecT was performed using equivalent settings to the above for the SEQUEST search.

InsPecT was performed in blind search mode (i.e. MSAlignment) allowing a maximum of two unanticipated modifications up to a maximum mass of 300 Da. As InsPecT in blind search mode is very time consuming, the search was performed using a small database containing only the known 18 standard proteins [21], common contaminants (e.g. trypsin and keratin) and an additional 100 proteins randomly chosen from the human database to act as distracter proteins.

## Availability and requirements

The algorithm, called msmsEval, is implemented in C++ and can be compiled for Windows, Linux or MacOS X using a standard GNU C++ compiler. msmsEval currently accepts files in mzXML file format [29] and outputs a file containing a summary of all MS/MS scans with their respective *p*(*D*|+) and identifiable distribution percentile values. There are options to generate filtered spectra in DTA format for convenient further analysis. On a Pentium Xeon 3.4Ghz processor, a typical analysis of a dataset containing 10,000 scans will take less than one minute. msmsEval is open-source and available for download at . Documentation and a tutorial with practical information on the usage of msmsEval are available at the website.

## Abbreviations

MudPIT – multidimensional protein identification technology

COFRADIC – combined fractional diagonal chromatography

LC^*n *^– multidimensional liquid chromatography

QDA – quadratic discriminant analysis

LDA – linear discrimiant analysis

## Authors' contributions

JWHW developed and implemented the algorithm and drafted the manuscript. MJS performed the annotation of the UCD dataset. HMC participated in the design of the algorithm. GC conceived the study, and participated in its design and helped to draft the manuscript. All authors read and approved the final manuscript.

## Supplementary Material

Additional file 1 **Supplementary figure 1**. Venn diagram showing the breakdown of successful spectrum annotations for the UCD dataset by different strategies. Click here for file

Additional file 2 **Appendices**. Contains details referred to in the manuscript regarding, 1) denoising of training datasets with k-nearest neighbor procedure, 2) comparison of classification procedures and 3) feature selection for the discrimination model. Click here for file

Additional file 3 **Supplementary figure 2**. Predicted distributions of identifiable and unidentifiable for a sample from the UCD test dataset (A) and sample A1 from the ISB dataset (B). The number of spectra is calculated with spectra placed in bins of 0.25 for the discriminant score. Spectra with fewer than five peaks are removed in line with the EM-algorithm. The solid line represents the actual distribution of spectra of the complete dataset and the dotted line represents the estimated distributions of identifiable and unidentifiable spectra using the expectation-maximization algorithm. Click here for file

## References

[B1] Aebersold R, Mann M (2003). Mass spectrometry-based proteomics. Nature.

[B2] Wolters DA, Washburn MP, Yates JR (2001). An automated multidimensional protein identification technology for shotgun proteomics. Anal Chem.

[B3] Gevaert K, Goethals M, Martens L, Van Damme J, Staes A, Thomas GR, Vandekerckhove J (2003). Exploring proteomes and analyzing protein processing by mass spectrometric identification of sorted N-terminal peptides. Nat Biotechnol.

[B4] Eng JK, McCormack AL, Yates JR (1994). An Approach to Correlate Tandem Mass Spectra Data of Peptides with Amino Acid Sequences in a Protein Database. J Am Soc Mass Spectrom.

[B5] Craig R, Beavis RC (2004). TANDEM: matching proteins with tandem mass spectra. Bioinformatics.

[B6] Perkins DN, Pappin DJ, Creasy DM, Cottrell JS (1999). Probability-based protein identification by searching sequence databases using mass spectrometry data. Electrophoresis.

[B7] Falkner J, Andrews P (2005). Fast tandem mass spectra-based protein identification regardless of the number of spectra or potential modifications examined. Bioinformatics.

[B8] Parekh RB, Rohlff C (1997). Post-translational modification of proteins and the discovery of new medicine. Curr Opin Biotechnol.

[B9] Bern M, Goldberg D, McDonald WH, Yates JR (2004). Automatic Quality Assessment of Peptide Tandem Mass Spectra. Bioinformatics.

[B10] Flikka K, Martens L, Vandekerckhove J, Gevaert K, Eidhammer I (2006). Improving the reliability and throughput of mass spectrometry-based proteomics by spectrum quality filtering. Proteomics.

[B11] Moore RE, Young MK, Lee TD (2000). Method for screening peptide fragment ion mass spectra prior to database searching. J Am Soc Mass Spectrom.

[B12] Nesvizhskii AI, Roos FF, Grossmann J, Vogelzang M, Eddes JS, Gruissem W, Baginsky S, Aebersold R (2006). Dynamic Spectrum Quality Assessment and Iterative Computational Analysis of Shotgun Proteomic Data. J Proteome Res.

[B13] Salmi J, Moulder R, Filen JJ, Nevalainen OS, Nyman TA, Lahesmaa R, Aittokallio T (2006). Quality classification of tandem mass spectrometry data. Bioinformatics.

[B14] Xu M, Geer LY, Bryant SH, Roth JS, Kowalak JA, Maynard DM, Markey SP (2005). Assessing data quality of peptide mass spectra obtained by quadrupole ion trap mass spectrometry. J Proteome Res.

[B15] Keller A, Nesvizhskii AI, Kolker E, Aebersold R (2002). Empirical statistical model to estimate the accuracy of peptide identifications made by MS/MS and database search. Anal Chem.

[B16] Nesvizhskii AI, Keller A, Kolker E, Aebersold R (2003). A statistical model for identifying proteins by tandem mass spectrometry. Anal Chem.

[B17] Tanner S, Shu HJ, Frank A, Wang LC, Zandi E, Mumby M, Pevzner PA, Bafna V (2005). InsPecT: Identification of posttranslationally modified peptides from tandem mass spectra. Anal Chem.

[B18] Tsur D, Tanner S, Zandi E, Bafna V, Pevzner PA (2005). Identification of post-translational modifications by blind search of mass spectra. Nat Biotechnol.

[B19] Han Y, Ma B, Zhang K (2005). SPIDER: software for protein identification from sequence tags with de novo sequencing error.. J Bioinform Comput Biol.

[B20] Brodley CE, Friedl MA (1999). Identifying Mislabeled Training Data. J Artif Intell Res.

[B21] Keller A, Purvine S, Nesvizhskii AI, Stolyar S, Goodlett DR, Koler E (2002). Experimental Protein Mixture for Validating Mass Spectral Analysis. OMICS: A Journal of Integrative Biology.

[B22] Tang XJ, Boyd RK (1992). An investigation of fragmentation mechanisms of doubly protonated tryptic peptides. Rapid Commun Mass Spectrom.

[B23] Hansen BT, Jones JA, Mason DE, Liebler DC (2001). SALSA: A pattern recognition algorithm to detect electrophile-adducted peptides by automated evaluation of CID spectra in LC-MS-MS analyses. Anal Chem.

[B24] Dempster AP, Laird NM, Rubin DB (1977). Maximum likelihood from incomplete data via the em algorithm. J Royal Stat Soc.

[B25] Duda RO, Hart PE, Stork GS (2000). Pattern Classification.

[B26] Cagney G, Park S, Chung C, Tong B, O'Dushlaine C, Shields DC, Emili A (2005). Human Tissue Profiling with Multidimensional Protein Identification Technology. J Proteome Res.

[B27] Frank A, Pevzner P (2005). PepNovo: De novo peptide sequencing via probabilistic network modeling. Anal Chem.

[B28] Sadygov RG, Eng J, Durr E, Saraf A, McDonald H, MacCoss MJ, Yates JR (2002). Code developments to improve the efficiency of automated MS/MS spectra interpretation. Journal of Proteome Research.

[B29] Institute of System Biology S Sashimi project. http://sashimi.sourceforge.net.

