# Supplementary material for: msmsEval: tandem mass spectral quality assignment for high-throughput proteomics
Source: BMC Bioinformatics. 2007 Feb 9;8:51. doi: 10.1186/1471-2105-8-51 (PMC1803797; doi:10.1186/1471-2105-8-51)
Supplement: Additional file 2 — Appendices. Contains details referred to in the manuscript regarding, 1) denoising of training datasets with k-nearest neighbor procedure, 2) comparison of classification procedures and 3) feature selection for the discrimination model. [file 1471-2105-8-51-S2.doc]

**SUPPLEMENTARY MATERIAL**

**Appendix 1.**

***Denoising of training datasets with k-nearest neighbor procedure***

To demonstrate the effect of denoising of a training dataset by the *k*-nearest neighbor (*k-*NN) algorithm, the ISB dataset is used as an example by assigning as positive those spectra originally found by Sequest, while assigning as negative all other spectra, including those annotated by Tsur and coworkers (2005) (i.e. spectra successfully annotated by Tsur are treated as false negatives). A 9-fold cross-validation strategy was used for generating results. For each validation, the training set consisted of all the identified spectra and a unique random selection of 10% of the unidentified spectra. For each such training set, the remaining unidentified spectra were classified by the *k-*NN algorithm. In the *k-*NN algorithm, three is chosen as *k*. After all 9 cross-validations have been completed, unidentified spectra that were “nearer” to identified spectra on 8 or more occasions were treated as mislabeled and removed.

The table below shows the effect of removing mislabeled spectra. Prior to denoising the percentage of false negatives within the dataset is 4.1%. Following denoising by the *k*-NN algorithm, this is reduced to 1.1%. A number of true negative samples are also removed. However, this is only a small proportion of the total number of true negatives and therefore the overall quality of the dataset is still improved.

|  | **Before denoising** | **After denoising** |
| --- | --- | --- |
| **False Negative** | 1403 (4.1%) | 377 (1.1%) |
| **True Negative** | 32915 (95.9%) | 27284 (98.9%) |
| **Total Negative** | 34318 | 27661 |

**Appendix 2**

***Comparison of classification procedures***

The percentage of identified and unidentified spectra from the UCD test dataset classified correctly. Linear discriminant analysis (LDA) and quadratic discriminant analysis (QDA) were trained using a subset from the UCD training dataset where all identified spectra and an equal number of unidentified spectra were randomly selected to form the final training set. In both the training and test samples, the spectra features were log transformed and mean centered. The decision boundary was chosen at the point that minimizes the total classification error in the training dataset.

The table below shows the percentage of spectra that were correctly classified as identified or unidentified by LDA and QDA. QDA in fact slightly under performs LDA in both cases, but the difference is not significant.

|  | **Correctly classified** | |
| --- | --- | --- |
| **Method** | **Identified** | **Unidentified** |
| **LDA** | 86.35% | 82.81% |
| **QDA** | 86.25% | 81.39% |

**Appendix 3**

***Feature selection for the discrimination model***

From the table below, it can be observed that *IntnRatio20%* and *H_2_ORatio* have t-probability values greater than the critical probability of 0.05. This indicates that these features do not contribute to the model significantly more than the null-hypothesis (i.e. that all spectra have the same value for that feature), inferring that these can be removed from the model.

In the table below, the coefficient, t-statistics and t-probabilities derived using logistic regression using all features available. The t-statistic is calculated by taking the ratio of the coefficient to its standard error. The t-probability level indicates the level at which the coefficient is significantly different to the null-hypothesis.

| **feature** | **coefficient** | **t-statistic** | **t-probability** |
| --- | --- | --- | --- |
| *NPeaks* | -0.005106 | -16.95 | <0.05 |
| *NormTIC* | 0.061926 | 4.47 | <0.05 |
| *GoodSegs* | 8.053273 | 43.15 | <0.05 |
| *IntnRatio1%* | -1.075026 | -9.34 | <0.05 |
| *IntnRatio20%* | -0.546333 | -1.70 | 0.08747 |
| *Complements* | 0.168857 | 30.59 | <0.05 |
| *IsoRatio* | 6.46695 | 22.78 | <0.05 |
| *H_2_ORatio* | -0.986949 | -1.56 | 0.1189 |
| *AAdiffRatio* | 7.470687 | 43.12 | <0.05 |
| *Constant (c_0_)* | -11.882207 | -59.52 | <0.05 |

The final table of coefficients used for this study is detailed below:

| **feature** | **coefficient** |
| --- | --- |
| *NPeaks* | -0.0055 |
| *NormTIC* | 0.0593 |
| *GoodSegs* | 3.9761 |
| *IntnRatio1%* | -1.1683 |
| *Complements* | 0.0813 |
| *IsoRatio* | 0.2004 |
| *AAdiffRatio* | 3.1107 |
| *Constant (c_0_)* | -7.9685 |
