# Supplementary figures and images for: msmsEval: tandem mass spectral quality assignment for high-throughput proteomics
Source: BMC Bioinformatics. 2007 Feb 9;8:51. doi: 10.1186/1471-2105-8-51 (PMC1803797; doi:10.1186/1471-2105-8-51)

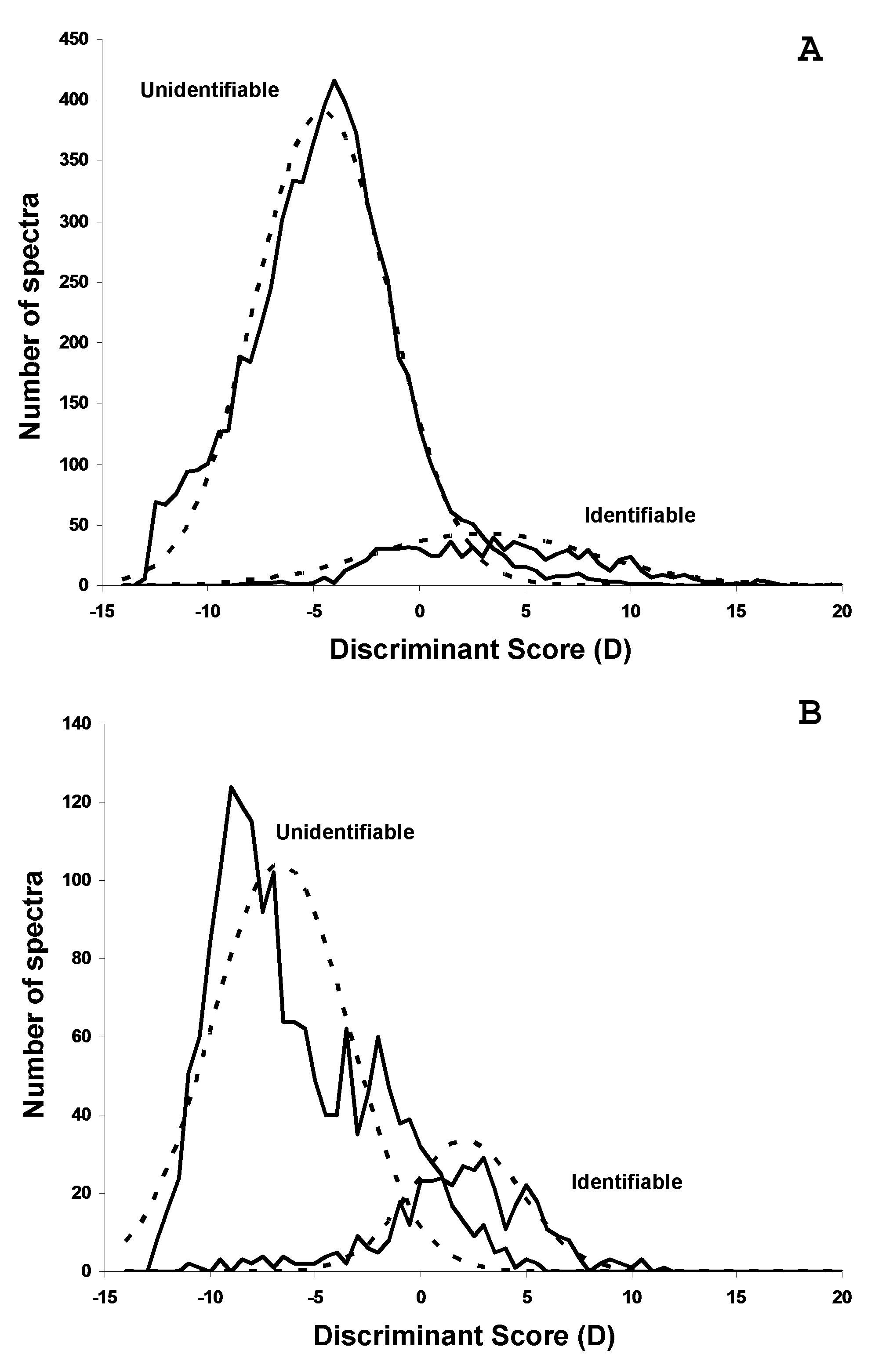

Supplement: Additional file 3 — Supplementary figure 2. Predicted distributions of identifiable and unidentifiable for a sample from the UCD test dataset (A) and sample A1 from the ISB dataset (B). The number of spectra is calculated with spectra placed in bins of 0.25 for the discriminant score. Spectra with fewer than five peaks are removed in line with the EM-algorithm. The solid line represents the actual distribution of spectra of the complete dataset and the dotted line represents the estimated distributions of identifiable and unidentifiable spectra using the expectation-maximization algorithm. [file 1471-2105-8-51-S3.tiff]
